# Precision medicine

**DOI:** 10.1515/medgen-2025-2028

**Published:** 2025-07-17

**Authors:** Christiane Zweier, Miriam Elbracht, Ilona Krey-Grauert

**Affiliations:** University Hospital Department of Human Genetics Freiburgstr. 15 3010 Bern Switzerland; Uniklinik RWTH Aachen Center for Human Genetics and Genomic Medicine Pauwelsstr. 30 52074 Aachen Germany; University of Leipzig Medical Center Leipzig Institute of Human Genetics Philipp-Rosenthal-Str. 55 04103 Leipzig Germany

An entire special issue on “Precision Medicine” highlights the clinical relevance the interdisciplinary field of human genetics now has in patient care. “Precision Medicine” or “Personalized Medicine” refers to tailored prevention and treatment measures that are based on the molecular, environmental, and lifestyle profile of a given individual. Understanding the molecular cause – and in many cases the genetic basis – of a disease is the key for translating this knowledge into targeted therapies.

The first major breakthroughs in precision medicine were achieved in oncology, a field in which treatment and surveillance based on characterization of tumors by genetic and other molecular biomarkers has been applied with increasing success in recent years.

However, also for many monogenic and/or rare diseases, higher diagnostic rates, and subsequently more specific counseling, prediction of prognosis, medical care, and treatment approaches, have also been achieved via advances in sequencing technologies and knowledge of pathomechanisms.

Two prominent non-oncological examples of diseases for which precision medicine has led to breakthroughs in patient care are Cystic Fibrosis (CF) and Spinal Muscular Atrophy (SMA). For CF, variant specific treatment is now available through CFTR modulation, while SMA can now be treated via antisense-oligonucleotides and gene therapy.

Our series of review articles is preceded by three case reports. These concern two patients treated for CF and SMA, respectively, and the parent of a child with a rare *GRIN2B*-related neurodevelopmental disorder. Though these are individual reports, they serve as examples of how new therapies can completely transform lives. The reports serve to motivate continued investment in basic and translational research in order to improve direct patient care. Clearly, benefits and costs cannot be measured on the basis of individual case reports. Instead, careful and systematic studies must be performed to demonstrate or to refute the impact of novel therapeutic approaches. However, the perspective of the patient must be an integral aspect of such investigations.

Four further contributions to this issue address different angles of the wide spectrum of precision medicine.

Jonas Käsbach and Jorgen Magnus present insights gained from biomedical engineering into the generation of Adeno-associated viruses (AAV), which represent the most widely applied vector system for *in vivo* gene therapy at the time of writing. They describe the development of AAV gene therapy from its initial inception to the present day. With increasing experience and an expanding number of clinical questions, the requirements for these therapies have become more diverse, and now include different strategies for optimization with respect to vector design, capsid engineering for tissue targeting, minimization of immunogenicity, and identification and reduction of process and product-related impurities. The authors review state-of-the-art AAV manufacturing technologies and platforms, and discuss their respective advantages and limitations in terms of issues such as efficacy and safety.

Ilona Krey, Irene Ferro, and Matias Wagner describe how antisense oligonucleotides (ASOs) are utilized as a RNA-targeting strategy to modulate gene expression in mostly rare or ultra-rare monogenic disorders. The article reviews approaches, selection criteria, and considerations for decision-making, as well as the respective limitations. The authors describe the development of the first ASO-therapies, and discuss both the successes and the challenges that have been encountered to date. Overall, they state that indication- and variant-dependent ASOs play an important role, in particular for the n-of-1 treatments, since design is also possible for individual patients with ultra-rare diseases. It remains to be seen what the long-term developments in this exciting field will be, and what major role human geneticists can play in the development and application of ASO therapies.

The article by Saranda Nimani, Miriam Barbieri, Marina Rieder, and Katja Odening reviews precision medicine from a cardiological perspective, using Long-QT-syndrome as an example. The authors discuss how genetic testing contributes to specific risk prediction for arrhythmias in clinical practice, as well as how different therapeutic strategies can be applied depending on the underlying molecular cause. To date, modulations of the respective ion channel functions have played the largest role. However, new gene therapies, particularly the application of hybrid Suppression-and-replacement (SupRep) gene therapy techniques, are a growing approach in this patient cohort.

Finally, the article by Ursula Amstutz addresses the important field of pharmacogenetics in patient care. Drug metabolism is highly dependent on pharmacogenetic characteristics, and knowledge of these characteristics can facilitate the optimization of pharmacotherapy with the aim of reducing adverse effects and improving effectiveness. Pharmacogenetics is one major aspect of precision medicine, and is a field that could be expanded in the future to the whole population. Pharmacogenetics also benefits from the increasing use of large-scale sequencing techniques, such as whole genome sequencing, and the increasing availability of bioinformatic tools for the analysis of pharmacogenetically-relevant genotype data. In the near future, the comprehensive analysis of relevant pharmacogenetic characteristics together with monogenic or polygenic causes of disease will optimize precision medicine for patients with genetic diseases.

Above all, human genetics is acknowledged to be an essential component of successful interdisciplinary precision medicine. Individual clinical treatment and preventive care are optimized when the genetic and other molecular mechanisms of disease development are understood, and the characteristics of individual patients, e. g., pharmacogenetically relevant genotypes, are taken into account.

